# Rollover Car Crashes with Ejection: A Deadly Combination—An Analysis of 719 Patients

**DOI:** 10.1155/2014/250540

**Published:** 2014-02-16

**Authors:** Rifat Latifi, Ayman El-Menyar, Hany El-Hennawy, Hassan Al-Thani

**Affiliations:** ^1^Trauma Surgery Section, Department of Surgery, Hamad Medical Corporation, P.O. Box 3050, Doha, Qatar; ^2^Department of Surgery, University of Arizona, P.O. Box 245063, Tucson, AZ, USA; ^3^Weill Cornell Medical College, P.O. Box 24144, Doha, Qatar; ^4^Clinical Research, Trauma Surgery Section, Hamad General Hospital, P.O. Box 3050, Doha, Qatar

## Abstract

Rollover car crashes (ROCs) are serious public safety concerns worldwide. *Objective*. To determine the incidence and outcomes of ROCs with or without ejection of occupants in the State of Qatar. *Methods*. A retrospective study of all patients involved in ROCs admitted to Level I trauma center in Qatar (2011-2012). Patients were divided into Group I (ROC with ejection) and Group II (ROC without ejection). *Results*. A total of 719 patients were evaluated (237 in Group I and 482 in Group II). The mean age in Group I was lower than in Group II (24.3 ± 10.3 versus 29 ± 12.2; *P* = 0.001). Group I had higher injury severity score and sustained significantly more head, chest, and abdominal injuries in comparison to Group II. The mortality rate was higher in Group I (25% versus 7%; *P* = 0.001). Group I patients required higher ICU admission rate (*P* = 0.001). Patients in Group I had a 5-fold increased risk for age-adjusted mortality (OR 5.43; 95% CI 3.11–9.49), *P* = 0.001). *Conclusion*. ROCs with ejection are associated with higher rate of morbidity and mortality compared to ROCs without ejection. As an increased number of young Qatari males sustain ROCs with ejection, these findings highlight the need for research-based injury prevention initiatives in the country.

## 1. Introduction

Rollover car crashes (ROCs) are associated with high morbidity and mortality, which is a serious public safety concern worldwide. While injuries associated with ROCs can range from minor to severe, injuries associated with ejection due to rollover are specifically severe. A recent study reported a 20-fold increased risk of severe injuries and 91 times greater risk of mortality if an individual is ejected completely from a car crash during an ROC [1]. Accordingly, fatality could be reduced by 70%, if the passengers of the motor vehicles are prevented from sustaining an ejection [2]. Kahane [3] reported that, even in less severe ROCs, two-thirds of the mortalities are attributed to occupant ejection from the vehicle. Also, ejection is a significant source of mortality among children [4].

The severity of injuries are usually determined by several factors, which include vehicle type, precrash speed, restraints (seat-belt) used, number of turns, intensity of the impact, vehicle damage, especially roof intrusion in relation to survival space, single or multivehicle event, type of rollover initiation, vehicle design, and field triage models [2, 5–11]. Age of the occupant, occupant size (BMI), location of occupant in the vehicle, and whether occupant was ejected or confined are also important parameters [9, 11]. Injuries during ejection might occur due to contact with the vehicle and/or ground. For example, Berg et al. [12] reported that ejected occupants sustained torso and head injuries mainly due to secondary impact with the ground or by being entrapped.

Ejection is associated with the most serious consequences in motor vehicle crashes (MVCs) and is mainly caused due to noncompliance of seat belt [13]. The majority of injuries due to ejection are usually preventable by the simple act of wearing a seat belt. Eigen [14] found 70% compliance of seat belts among injured nonejected occupants. On the other hand, 51% of the partially ejected and only 3% of completely ejected occupants used restraints. When estimating the effects of wearing a seat belt, Winnicki [13] reported that controlling ejection of drivers significantly reduces the rate of morbidity and mortality by 58% and 72%, respectively.

Qatar ranks high on fatalities due to road traffic accidents (RTAs) [15]. Particularly, MVCs are more prevalent among the young Qatari nationals as opposed to the other nationalities residing in Qatar. A study based on Driver Behavior Questionnaire (DBQ) found that the majority of Qatari national drivers who were involved in traffic violations were under the age of 30 years [16]. The study also observed more aggressive behaviors while driving. Therefore, factors involving increased risk of ejection need to be analyzed for controlling significant risk of injury and mortality in ROCs with ejection. The present study aimed to identify the factors contributing to ejection during ROCs and to provide insight into public policy efforts that can reduce the risk of ejection within the country of Qatar. Our goal is to empirically test the effects of ejection from the vehicle on morbidity and mortality outcomes as well as to analyze the predictors that facilitate ejection.

As suggested by the literature, we hypothesized that individuals ejected during ROCs sustain higher rates of morbidity and mortality than those who were not ejected. These results will be reflected in patient outcomes as admitted to Intensive Care Unit (ICU) and those who died. We also hypothesize that specific demographic characteristics might contribute to the likelihood of an ejection event. Mainly, age and nationality will predict the use of seat belt compliance.

## 2. Methods

A retrospective review was conducted of all patients involved in ROCs with or without ejection admitted to a Level I trauma center, Hamad General Hospital (HGH) in Qatar, during January 2011 and December 2012. Demographics (age, gender, and nationality), passenger location in vehicle, use of protective devices, mode of transportation to the trauma center, injury severity score (ISS), type of injuries, anatomical body parts injured, ethanol intake, hospital length of stay (LOS), intensive care unit (ICU) LOS, morbidity, and mortality were analyzed. Patients' demographics, clinical presentation, type of injuries, position in the vehicle, protective devices used, complications, and mortality were analyzed.

Data were also analyzed according to whether the occupant was ejected (Group I) or not (Group II) to ascertain the effect of ROCs with and without ejection on demographic factors and patient outcomes.

Data were presented as proportions, mean ± standard deviation (SD), or median and range whenever applicable. Demographic factors and patient outcomes were analyzed according to ROC with and without ejection using the student's *t*-test for continuous variables and Pearson chi-square (*χ* ^2^) test for categorical variables. For skewed continuous variables, nonparametric test was performed. Multivariate logistic regression analysis was performed after adjusting for age to see whether ejection is a predictor for mortality. Correlation between ejection and ISS was determined using side-to-side boxplot. A significant difference was considered when the 2-tailed *P* value was less than 0.05. Data analysis was carried out using the Statistical Package for Social Sciences version 18 (SPSS Inc., USA). This study has been approved by the medical research center at Hamad Medical Corporation, Qatar (IRB number 13123/13).

## 3. Results

**Figure 1:**
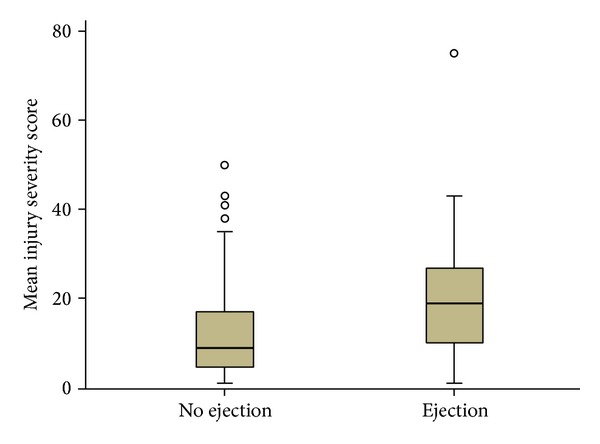
Side-to-side boxplot for correlation of rollover with ejection and injury severity score.

A total of 719 patients were involved in ROCs, of them 89.6% were males with mean age of 27.5 ± 11.9 years (Table 1). While natives of Qatar represent approximately 18% of the population, the percentage of Qatar nationals involved in ROCs in our study was found to be 41%. Among ROCs, the majority of victims were drivers (54%) followed by front (15.6%) and back (15.3%) seat passengers. Ejection of occupants from the motor vehicle was reported in 33% cases (Table 1). The overall compliance of protective measures was very low: only 16% occupants used seat belts and also protection by airbag was documented in only 1% of crashes. Scene intubation was required in 31% cases. Chest (42%), head (36%), spine (34%), and upper extremity (32%) were the most frequently injured body parts. The mean ISS was 14.7 ± 10.6; respective AIS for head, chest, and abdomen was 3.4 ± 1, 2.8 ± 0.6, and 2.5 ± 0.8, respectively (Table 1). The median length of stay in ICU was 4 (range 1–155) days and the overall hospital stay was 6 (range 1–368) days.Table 2 shows the demographic factors and patient outcomes according to whether the occupant was ejected or not in ROCs. The age of individuals ejected from the vehicle was significantly younger than the nonejected individuals (24.3 ± 10.3 versus 29 ± 12.2 years, *P* = 0.001). The incidence of ROC with ejection was significantly higher among Qatari nationals, as compared to ROC without ejection (54.8% versus 34.3%; *P* = 0.001). Also, higher rates of ejected occupants were drivers (63% versus 50%; *P* = 0.001). Interestingly, none of the ejected occupants of the vehicles were wearing a seat belt. The rate of scene intubation (57% versus 18%; *P* = 0.001) was higher in ejected individuals with lower scene GCS (10.6 ± 5 versus 13.7 ± 3.3; *P* = 0.001) in comparison to nonejected individuals. The rate of ejected patients needing ICU admission was significantly higher among patients ejected than nonejected (50% versus 22.3%; *P* = 0.001). Also, ejected patients were more severely injured with greater ISS (20 ± 12 versus 12 ± 9; *P* = 0.001) and sustained more head (53.6% versus 26.8%; *P* = 0.001), chest (52.3% versus 36.7%; *P* = 0.001), and abdominal (25.7% versus 16.6%; *P* = 0.003) injuries than nonejected patients. Figure 1 shows side-to-side Boxplot for the correlation between ROCs with ejection and the higher mean ISS.

The median ICU (6 (range 1–155) versus 3 (range 1–90); *P* = 0.04) and hospital stay (8.5 (1–192) versus 6 (1–368); *P* = 0.02) were longer among ejected patients than those not ejected.

The overall mortality of patients in this study was 12.7%; the rate of mortality (25% versus 7%; *P* = 0.001) was also higher in ejected patients as compared to nonejected patients. After adjusting for age, ejection was an independent predictor for mortality (odd ratio 5.43; 95% CI 3.11–9.49, *P* = 0.001).

## 4. Discussion

This is a unique study that analyzes the various factors of ROCs in Qatar and adds to the body of literature that has recognized that ROCs with ejection is a deadly combination. The present study identifies the incidence and outcome of ROCs in Qatar with particular focus on demographic characteristics and patient outcomes. Furthermore, this study further confirms that ejection from a motor vehicle is associated with increased incidence of morbidity and mortality. Earlier studies from Qatar suggested a high incidence of MVCs and involvement of traffic violations [15, 16]. The State of Qatar has a population of almost 1.7 million [17] of which 60% of the population reside in Doha. The majority of the population is expatriates from South-East Asia, North Africa, Levant Arab countries, and East Asia. HGH is the only Level I trauma center in Qatar and it sees and admits more than 98% of all victims of trauma in Qatar. Thus, the findings of this study are truly representative of ROCs in Qatar. The majority of victims of ROCs in Qatar are young males. These victims are Qatari national (41%) and non-Arab population (39%). Our findings are corroborated by earlier reports, which also found a higher involvement of RTAs and risk taking behavior while driving among Qatari drivers [15, 16, 18]. Also, a recent study on drivers behavior among different ethnic groups in Qatar reported that Qatari drivers are at increased risk for MVC, which is in contrast with Europe where the immigrant population is considered at greater risk of MVC in comparison to the national population [16].

There are many factors that affect the outcome of ROCs. The kinematics of vehicle occupants is considerably affected by the direction of the rollover, magnitude of forces, and position of the occupant [19]. In our study, the majority of the victims were drivers (54%) and front seat (15.6%) passengers. This is similar to Atkinson et al. [20] who reported a significantly higher rate of ejection among drivers in comparison to other passengers. Among unrestrained occupants, 62% of drivers and 51% of front seat passengers experienced ejection. Also, the rate of ejection for near-sided and far-sided passengers was comparable to our study. Parenteau et al. [21] reported that occupants of farside sustained serious injuries compared to nearside occupants. They also concluded that head injury was more common among drivers (farside) than nearside passengers. Occupants of far side sustained spine, head, and thorax injuries. It has been well established that seat belts are highly effective in preventing complete ejection of occupants from vehicles during various crashes as a seat belt provides appropriate restraining force.

The risk of partial ejection could be minimized to a greater extent by seat belt use [22]. According to National Crash Analysis Center, USA, data from 1995–2001, the majority (75%) of the front seat passengers who experienced ROCs were using seatbelts. The rate of ejection in this study was very low, that is, only 0.2% for the belted and 4.2% for the unbelted occupants. Moreover, the rate of injury for complete ejection was 12-fold higher in comparison to nonejected occupants [2]. The overall compliance of protective measures in our study was extremely low, with only 16% of occupants using seat belts. The unrestrained occupants experience severe injuries due to intrusion by rollover in majority of the cases [9]. Complex injuries of head/neck and hemo/pneumothoraxes are the most significant injuries sustained during rollover car crashes. Mandell et al. [23] showed that with an increase in degree of roof crush in ROCs, the risk of mortality and severe head/spinal injuries also increases significantly. However, the nonejected occupants experienced head, spine, and extremities injuries primarily by roof crush or impact of the interior on the occupant [12]. But the severity of head/neck injuries among unrestrained passengers who experienced ejection does not depend only upon roof strength [24]. Funk et al. [1] observed an increased risk of severe and fatal injuries of head and cervical spine among ROCs with complete or partial ejection, noncompliance of seat belt, higher numbers of roof inversions, and advanced age of occupants. These findings further support our hypothesis that ejection is associated with worse outcomes. Injuries of head, chest, and abdomen were significantly higher for ejected occupants versus nonejected. More than half of ejected individuals sustained head injuries, whereas only 26.8% of nonejected individuals sustained head injuries. According to anatomic body regions, head sustained the most severe injuries (AIS+3), but they are nonsignificantly higher for ejected (3.5 ± 1) as well as nonejected (3.3 ± 0.9) occupants in our study. Our findings are supported by another recent study, which also observed an increased rate of serious injury based on rollover severity among ejected as well as nonejected occupants [1].

It has been shown that the age of the driver is an important predictor of probability for an ejection event in ROCs. An earlier NHTSA report demonstrated that younger drivers are more susceptible to ejection crashes. The mean age of drivers involved in ROCs was 33 years, whereas the mean age of ejected driver in ROCs was 32 years [25]. The present study also found an increased association of young age with ejection. This is similar to other studies where the mean age of individuals who experienced ROC was 28 years, while the mean age of ejected occupants in ROC was even much lower (24 years), although in our study there was a difference between those who were ejected and who were those ejected (24.3 ± 10.3 versus 29 ± 12.2; *P* = 0.001). In a rollover, there is a fivefold increased risk of mortality, if the occupant was ejected during the crash. It was also suggested that the fatality rate could be reduced by 70% by effective controlling of ejection in rollover [2]. Another study reported that, even in less severe ROCs, two-thirds of the mortalities were attributed to occupant ejection from the vehicle [3]. Deutermann [26] found ejection as the primary cause of mortality in 62% of severe ROCs. According to the Advanced Glazing Project of NHTSA, which analyzed ejection ROCs, a relative risk of fatality for ejected to nonejected occupants was found to be 3.55 for drivers and 3.15 for front seat passengers. So, controlling ejection of drivers significantly helps in reducing the rate of morbidity by 58% and mortality by 72%, respectively [13].

ROCs are a major cause of morbidity and mortality amongst children. Howard et al. [27] reported that ejection from the vehicle is frequently associated with mortality (29%) among young children. Further, infants and young children (0 to 4 years) had the highest fatality rate and were mostly found to be either unrestrained or improperly restrained. The relative risk of mortality in ejected occupants ranged from about 1.5 to 8 depending upon the crash mode or type. Single-vehicle ROCs with ejection have the highest risk of mortality [28]. Also in our study the rate of mortality was significantly higher among ejected individuals (24.5%) as compared to (6.8%) nonejected individuals. Therefore, factors involving increased risk of ejection need to be analyzed for controlling significant risk of injury and mortality in ROCs with ejection. The strength of this empirical evaluation it that it shows significant differences in patient outcomes for individuals who have been in ROCs whether they were ejected or not ejected from the vehicle. It is well known that the highest rates of morbidity and mortality are associated with ejection from the vehicle. An obvious preventable predictor of ejection during ROCs is wearing a restraint, such as a seat belt. With the obvious outcomes associated with ejection, this leads to the question of why all individuals do not wear seat belts.

The present study has some limitations as with any other retrospective analysis of data. Since it is a registry-based analysis, information regarding precrash speed, number of turns, intensity of the impact, vehicle damage especially roof intrusion in relation to survival space, single or multivehicle event, type of rollover initiation, vehicle design, and farside and nearside impact are not available.

Furthermore, those who died at the scene often do not arrive to our trauma center at all and were not included in this study. A prospective study has already been initiated that involves multidisciplinary approaches (trauma, EMS, and police) to investigate the multidimensional and multi factorial issues (driver, car, and road related factors) which are lacking in this retrospective study for specific injury prevention programs to prevent ROC in Qatar.

In summary, ROCs with ejection are associated with higher rate of morbidity and mortality. In our study, an increased incidence of ROCs has been observed particularly among young Qatari males. These findings highlight the need for research-based injury prevention initiatives to assess the societal and individual influences that contribute to practices of aggressive driving and noncompliance of safe measures. Furthermore, it may highlight the need for better look at the road engineering as a major factor. Our data provide an empirical opportunity to assess the outcomes associated with ejection from vehicles. An additional insight may be provided by patient interviews, if possible, after admission. The questions that still need to be addressed are why individuals do not use seat belts when seat belt usage is an obvious protective factor against fatality and serious injury and law requires it. Such law, however, is not currently reenforced appropriately. There are complex interactions between policy makers, system-wide institutions, transitions (such as that experienced in the rapid transition experienced in the country of Qatar in the last decade), and individual decision-making [29]. Our findings also have serious implications for education and training of drivers involved in rash driving as well as for safety campaigns.

## Figures and Tables

**Table 1:** Overall analysis of rollover car crashes (ROCs) (*n* = 719).

Age (mean ± SD)	27.5 ± 11.9
Male	644 (89.6%)
Qataris	282 (40.8%)
Position in vehicle	
Driver	382 (53.1%)
Front passenger	110 (15.3%)
Back passenger	108 (15.0%)
Unspecified	106 (15%)
Ejected	237 (33%)
Protective devices used	
Seatbelt	113 (15.7%)
Airbag	8 (1.1%)
Mode of transport	
Ambulance	576 (81%)
Helicopter	115 (16.2%)
Private vehicle	13 (1.8%)
Private ambulance	7 (1%)
Scene intubation	223 (31%)
Associated injuries	
Chest	301 (41.9%)
Head	256 (35.6%)
Spine	247 (34.4%)
Abdomen	141 (19.6%)
Pelvis	114 (15.9%)
Upper extremity	233 (32.4%)
Lower extremity	149 (20.7%)
Head AIS (mean ± SD)	3.4 ± 1
Chest AIS (mean ± SD)	2.8 ± 0.6
Abdominal AIS (mean ± SD)	2.5 ± 0.8
ISS (mean ± SD)	14.7 ± 10.6
GCS scene	12.7 ± 4.2
ED LOS (median; range)	4.8 (<1–77)
ED disposition	
Operating room	97 (13.6%)
ICU	224 (31.5%)
Surgical floor	338 (47.5%)
Died in ED	46 (6.5%)
Transferred*	7 (1%)
ICU LOS (days) (median; range)	4 (1–155)
Hospital LOS (days) (median; range)	6 (1–368)
Blood transfusion	136 (18.9%)
Blood units (median; range)	4 (1–39)
Ethanol mmol/L (mean ± SD)	35.2 ± 18.1
Mortality	91 (12.7%)

GCS: glasgow coma score; ED: emergency department; LOS: length of stay; OR: operation room; ICU: intensive care unit; AIS: abbreviated injury score; ISS: injury severity score; *only burn patients are transferred to another hospital in the campus, the burn center.

**Table 2:** Comparative analysis of rollover car crashes (ROCs) with and without ejection.

	ROCs with ejection (Group I = 237)	ROCs without ejection (Group II = 482)	*P* value
Age (mean ± SD)	24.3 ± 10.3	29 ± 12.2	0.001
Male (%)	90.7	89	0.29
Nationality			
Qatari (%)	54.8	34.3	0.001 for all
Non-Qatari (%)	45.2	65.7	
Position in vehicle			
Driver (%)	63.2	49.6	0.001 for all
Front passenger (%)	12	17.4	
Back passenger (%)	9.4	18.2	
Unspecified (%)	15.4	14.8	
Mode of transport			
Ambulance (%)	77	83	0.003 for all
Helicopter (%)	22.1	13.2	
Private vehicle (%)	0.9	2.3	
Private ambulance (%)	0.0	1.5	
Protective devices used			
Seatbelt (%)	0.0	23.4	0.001
Airbag (%)	0.8	1.2	0.48
Intubation (%)	57.4	18	0.001
GCS scene (mean ± SD)	10.6 ± 5	13.7 ± 3.3	0.001
ED LOS (hours) (median; range)	2.6 (<1–50)	5.7 (<1–77)	0.001
ED disposition (%)			
ICU	50.0	22.3	0.001 for all
Operating room	16.1	12.4	
Surgical floor	23.7	59.2	
Died in ED (%)	10.2	4.6	
Transferred	0	1.5	
Associated injuries			
Head (%)	53.6	26.8	0.001
Chest (%)	52.3	36.7	0.001
Abdomen (%)	25.7	16.6	0.003
Spine (%)	35.9	33.6	0.30
Pelvis (%)	25.7	11	0.001
Upper extremity (%)	36.7	30.3	0.05
Lower extremity (%)	22.4	19.9	0.25
Head AIS (mean ± SD)	3.5 ± 1	3.3 ± 0.9	0.15
Chest AIS (mean ± SD)	2.9 ± 0.6	2.7 ± 0.7	0.001
Abdominal AIS (mean ± SD)	2.7 ± 0.9	2.4 ± 0.7	0.01
ISS (mean ± SD)	20.1 ± 11.8	12.2 ± 8.9	0.001
ICU LOS (days) (median; range)	6 (1–155)	3 (1–90)	0.04
Hospital LOS (days) (median; range)	8.5 (1–192)	6 (1–368)	0.02
Blood transfusion (%)	33.8	11.6	0.001
Ethanol mmol/L (mean ± SD)	34.8 ± 15.5	35.5 ± 20.4	0.89
Mortality (%)	24.5	6.8	0.001

GCS: glasgow coma score; ED: emergency department; LOS: length of stay; OR: operation room; ICU: intensive care unit; AIS: abbreviated injury score; ISS: injury severity score.
